# Suppression of Selective Voltage-Gated Calcium Channels Alleviates Neuronal Degeneration and Dysfunction through Glutathione S-Transferase-Mediated Oxidative Stress Resistance in a *Caenorhabditis elegans* Model of Alzheimer's Disease

**DOI:** 10.1155/2022/8287633

**Published:** 2022-11-30

**Authors:** Zihui Zheng, Kanglu Wu, Qinli Ruan, Dongfang Li, Weizhen Liu, Min Wang, Yaoyao Li, Jintao Xia, Dongqing Yang, Jun Guo

**Affiliations:** School of Medicine & Holistic Integrative Medicine, Nanjing University of Chinese Medicine, Nanjing 210023, China

## Abstract

Calcium homeostasis plays a vital role in protecting against Alzheimer's disease (AD). In this study, amyloid-*β* (A*β*)-induced *C. elegans* models of AD were used to elucidate the mechanisms underlying calcium homeostasis in AD. Calcium acetate increased the intracellular calcium content, exacerbated A*β*_1–42_ aggregation, which is closely associated with oxidative stress, aggravated neuronal degeneration and dysfunction, and shortened the lifespan of the *C*. *elegans* models. Ethylene glycol tetraacetic acid (EGTA) and nimodipine were used to decrease the intracellular calcium content. Both EGTA and nimodipine showed remarkable inhibitory effects on A*β*_1–42_ aggregations by increasing oxidative stress resistance. Moreover, both compounds significantly delayed the onset of A*β*-induced paralysis, rescued memory deficits, ameliorated behavioral dysfunction, decreased the vulnerability of two major (GABAergic and dopaminergic) neurons and synapses, and extended the lifespan of the *C. elegans* AD models. Furthermore, RNA sequencing of nimodipine-treated worms revealed numerous downstream differentially expressed genes related to calcium signaling. Nimodipine-induced inhibition of selective voltage-gated calcium channels was shown to activate other calcium channels of the plasma membrane (*clhm-1*) and endoplasmic reticulum (*unc-68*), in addition to sodium-calcium exchanger channels (*ncx-1*). These channels collaborated to activate downstream events to resist oxidative stress through glutathione S-transferase activity mediated by HPGD and *skn-1*, as verified by RNA interference. These results may be applied for the treatment of Alzheimer's disease.

## 1. Introduction

The prevalence of neurodegenerative diseases has continued to increase along with the ever-expanding elderly population. Alzheimer's disease (AD), a neurodegenerative disease with the highest morbidity, accounts for about 70% of dementia cases, and is estimated to affect 131.5 million people by 2050 given the current lack of effective therapies [1]. Numerous studies conducted over several decades have identified cholinergic deficiency, aggregation of amyloid-*β* (A*β*) peptides, tau protein hyperphosphorylation, synaptic dysfunction, oxidative stress, dysregulation of calcium signaling, and neuroinflammation as pathomechanisms of AD [2, 3]. Hence, understanding the interplay among different pathological factors and pathways is key to the development of a treatment strategy for this multifaceted disease.

The deposition of A*β* peptides is considered histopathological hallmark of AD. In particular, aggregates of A*β* peptides damage nerve cells through a variety of biological effects, including calcium steady-state imbalance and oxidative stress. A*β* aggregates promote activation of voltage- and receptor-gated calcium channels, while inhibiting calcium extrusion through plasma membrane calcium ATPase or sodium/calcium exchangers, resulting in elevated intracellular calcium levels and disruption of calcium homeostasis [4–8]. In addition, calcium dysregulation further aggravates A*β* deposition and is involved in multiple pathologic processes, such as impaired synaptic plasticity, neuronal damage, and apoptosis [9]. These findings indicate that inhibition of calcium influx could potentially prevent the progression of AD.

Oxidative stress contributes significantly to the pathogenesis and progression of AD. In the brain tissues of AD patients, the expression levels of many biomarkers of oxidative damage are elevated in the regions with rich A*β* plaques [10, 11]. A*β* plaque deposition can lower production of adenosine triphosphate (ATP) and increase production of reactive oxygen species (ROS) [12, 13]. Increased ROS production is reported to induce or mediate A*β*-induced brain cell senescence in a mouse model of AD and in cultured brain cells [14–16]. There is a positive feedback loop between A*β* plaque deposition and ROS production. In addition, increased ROS production has been associated with impairment of calcium influx through N-methyl-D-aspartate receptors in primary cortical neurons [17, 18] and elevated ROS production in mitochondria can be prevented by blocking calcium uptake [19]. It is likely that ROS-mediated disruption of calcium homeostasis promotes the progression of AD. Therefore, restoration of calcium homeostasis could impede the development of AD by increasing resistance to oxidative stress.

*Caenorhabditis elegans* (*C. elegans*) is highly amenable to genetic manipulation and presents a useful *in vivo* model to investigate the mechanisms of several human neurodegenerative diseases. *C*. *elegans* models of AD are available for expression of human A*β*_1-42_ protein aggregates in both neurons and muscle cells to assess behavioral and pathological changes. *C*. *elegans* has an evolutionarily conserved calcium signaling pathway similar to that of other animals [20]. In the present study, both *C*. *elegans* models of AD were used to explore the interplay between A*β* and intracellular calcium. Neurodegenerative behaviors, the vulnerability of two types of major neurons (gamma-aminobutyric acid and dopamine), and synapses in *C. elegans* were observed in response to the mutual influence of A*β* and calcium. The potential signaling pathways involved in ameliorating AD progression *via* calcium homeostasis were explored by RNA sequencing and RNA interference (RNAi).

## 2. Materials and Methods

### 2.1. C. Elegans Strains

The wild-type strain N2, the transgenic strains of BL5717 (*ida-1p*::GFP), CL2355 (*dvIs50*), CL4176 (*dvIs27*), CL2122 (*dvIs15*), CL802 (*cc546*), CL2166 (*gst-4p*::GFP::nls), EG1285 (*unc* − 47*p* : GFP + *lin* − 15(+)), UA57 (*dat* − 1*p* : GFP + *dat* − 1*p* : CAT − 2) NC571 (*unc* − 4*p* : *snb* − 1 : GFP + *dpy* − 20(+)), and mutant strain *him-5* (e1490), were obtained from the Caenorhabditis Genetics Center (University of Minnesota, Minneapolis, MN, USA). The detailed description about strains is shown in Supplementary Table S1.

### 2.2. Genetic Crosses

The transgenic strains NUCM0001, NUCM0002, NUCM0003, and NUCM0004 were generated by crossing strain CL2355 with strains EG1285, BL5717, UA57, and NC571, respectively. Strain CL2355 was developed as an AD model with a green fluorescent protein (GFP) marker in the gut. The transgenic strains EG1285, BL5717, UA57, and NC571 express GFP fusion proteins in GABAergic neurons, motor neurons, dopaminergic neurons, and synapses. Briefly, N2 males were mated with EG1285, BL5717, UA57, and NC571 worms, and the resulting transgenic male offspring then crossed with CL2355 hermaphrodites. The progenies with GFP expressed in both the gut and neurons were identified by fluorescence microscopy. Heterozygotes were separated from the homozygotes that were further examined for the presence of both transgenes and genotyped via single-worm PCR using a primer pair for human A*β* (forward: 5′-GAT GCA GAA TTC CGA CAT GA-3′; reverse: 5′-TCG CTA TGA CAA CAC CGC CCA-3′). The genotypes and phenotypes of these strains are listed in Supplementary Table S1.

### 2.3. C. Elegans Maintenance and Treatment

Worms were cultivated on nematode growth medium (NGM) plates and fed *Escherichia coli* strain OP50 in accordance with standard methods [21]. Gravid animals were washed off the plates into centrifuge tubes and then lysed with a bleaching mixture. Age-synchronized populations of L1 larvae were obtained by successively washing with double-distilled water and K medium, and maintained on NGM plates containing a vehicle, calcium acetate (2, 20, 100, and 200 mM), ethylene glycol tetraacetic acid (EGTA) (0.25, 2.5, 25, and 250 mM), or nimodipine (0.5, 1, 20, and 40 mM). About 100 animals were transferred to each NGM plate. Calcium acetate and EGTA were purchased from Sinopharm Chemical Reagent Co., Ltd. (Shanghai, China) and nimodipine was obtained from Shanghai Yuanye Bio-Technology Co., Ltd. (Shanghai, China).

### 2.4. Neurodegenerative Behavior Assays

#### 2.4.1. Paralysis Assay

Synchronized CL4176 L1 larvae were maintained at 16°C for 38 h on NGM plates containing a vehicle, calcium acetate, or EGTA. Transgenic expression was induced by increasing the temperature to 23°C and the paralysis phenotype was observed after 42 h. Scoring was performed hourly until the last worm became paralyzed. Paralysis was confirmed by the appearance of a transparent ring around the head and movement restricted to the head when gently touched with a platinum loop. Each experiment was performed with triplicate groups of at least 100 worms. Nimodipine was administrated 8 h before observation.

#### 2.4.2. Chemotaxis Assay

Chemotaxis was assessed with the odorant preference assay as described by Margie et al. [22]. Briefly, age-synchronized CL2355 L1 larvae were maintained on NGM plates containing a vehicle, calcium acetate, or EGTA at 16°C for 36 h, followed by 23°C for 36 h. Synchronized worms were harvested and assayed on 90-mm NGM plates, where 1 *μ*L of an odorant (0.1% benzaldehyde) and 1 *μ*L of 1 M sodium azide solution were applied to the “attractant” spot, while 1 *μ*L of the control odorant (100% ethanol) and 1 *μ*L of sodium azide were applied to the opposite side of the attractant spot. The chemotactic index (CI) was calculated as the difference between the number of worms in both attractant quadrants and the number of worms in both control quadrants divided by the total number of scored worms. Each experiment was conducted with three replicates. Nimodipine was administrated 8 h before observation.

#### 2.4.3. Analysis of Body Bends Capacity

CL2355 worms were treated as described in section 2.4.2. The frequency of body bend in 30 s was calculated on bacteria-free plates. To avoid overstimulation, each measurement was conducted at 5 min after transfer. Each experiment was performed using three replicates of at least 10 worms.

### 2.5. Lifespan Assay

CL4176 worms were treated as described in section 2.4.1. The mortality rate was calculated at 42 h after the incubation temperature was increased to 23°C. The mortality rate was scored every day until all worms had died. The fraction of surviving worms was calculated [23]. Each experiment was performed with three replicates of at least 100 worms.

### 2.6. Fluorescent Staining of A*β* Peptides with Thioflavin S (ThS)

Age-synchronized CL2355 L1 larvae were maintained at 16°C on NGM plates containing a vehicle, calcium acetate, or EGTA. After cultivation at 23°C, synchronized worms were harvested, fixed overnight, and stained with 0.125% ThS (Sigma-Aldrich Corporation, St. Louis, MO, USA) in 50% ethanol, and observed under a light microscope (Axio Scope.A1; Carl Zeiss AG, Jena, Germany) equipped with a charge-coupled device digital camera. The number of spots positive for ThS in the head region was counted. The data are expressed as the number of A*β* deposits in the anterior region. Each experiment was performed using three replicates of at least 50 worms. Nimodipine was administrated 8 hours before the observation.

### 2.7. Fluorescent Microscopy

#### 2.7.1. Visualization of Neurons and Synapses

Approximately, 30 in day 1 adult transgenic worms were mounted on agarose pads and paralyzed with 50 mM levamisole solution. Micrographs of GABAergic and dopaminergic neurons and synapses were acquired with a fluorescence microscope. Potential loss of D-type GABAergic motor neurons and dopaminergic neurons in transgenic worms was assessed by counting the number of cell bodies. Gaps in the ventral nerve cord (VNC) were quantified to reflect synapsis degeneration. Fluorescence intensity was analyzed with ZEN imaging software (Carl Zeiss AG).

#### 2.7.2. GFP Fluorescence Microscopy

Age-synchronized CL2166 L4 larvae were treated with 5 mM paraquat (PQ) as models of oxidative stress. At 6 h after PQ treatment, the larvae were exposed to nimodipine for 8 h, then fixed with 5 mM levamisole, and imaged under a fluorescence microscope.

### 2.8. ATP Measurement

ATP production was quantified using an ATP Assay Kit (Beyotime Institute of Biotechnology, Shanghai, China) as described by Zhang et al. [24]. Chemiluminescence detection of ATP was performed using a SpectraMax i3x microplate reader (Molecular Devices, LLC, San Jose, CA, USA). The ATP content was calculated from a calibration curve of standard solutions (0.01–10.0 *μΜ*) and normalized to the protein content.

### 2.9. ROS Measurement

The worms were collected into a microfuge tube at 38 h after the temperature was increased, washed three times with M9 buffer, and soaked in 50 *μ*M dihydroethidium (DHE) (ROS Assay Kit, Applygen Technologies Incorporation, Beijing, China) for 50 min without light. Then, the worms were washed with M9 buffer for three times, and examined for the fluorescent signals at excitation and emission wavelengths of 485 and 525 nm under a fluorescence microscope.

### 2.10. RNAi

Loss-of-function phenotypes were generated by RNAi. Following RNAi of *clhm-1*, *unc-68*, and *ncx-1*, CL2355 worms were fed *E. coli* strain HT115. RNAi treatment started from the first generation larva to the second generation adult. L1 larvae were incubated on plates containing RNAi or a control vector at 15°C for 36 h, and then at 23°C for 36 h. *E*. *coli* strain HT115 carrying the RNAi empty vector L4440 was used as a control. RNAi efficiency was determined by quantitative real-time polymerase chain reaction (qRT-PCR) with the primers listed in Supplementary Table S2.

### 2.11. RNA Isolation and qRT-PCR Analysis

Age-synchronized embryos were maintained on NGM plates seeded with bacteria at 16°C for 36 h and then at 23°C for 36 h. Total RNA was isolated with Beyozol reagent (Beyotime Institute of Biotechnology) and reverse-transcribed into complementary DNA using PrimeScript™ RT Master Mix (Takara Bio, Inc., Shiga, Japan). Transcript levels of the genes of interest were normalized to that of *act-3* as a control. The cDNA products were amplified by qRT-PCR using LightCycler® RNA Master SYBR Green I (Roche Life Science, Penzberg, Germany) and the primers listed in Supplementary Table S2.

### 2.12. RNA Sequencing

An RNA library was prepared from 1 *μ*g of total RNA with the TruSeq™ RNA sample preparation Kit (Illumina, Inc., San Diego, CA, USA). RNA sequencing was performed with the HiSeq Xten/NovaSeq 6000 sequencer (read length, 2 × 150 bp; Illumina, Inc.). The clean reads were individually aligned to the *C. elegans* genome (http://metazoa.ensembl.org/Caenorhabditis_elegans/Info/Index) using HISAT2 software (http://ccb.jhu.edu/software/hisat2/index.shtml) [25]. The levels of differentially expressed genes (*p* ≤ 0.05; log_2_ (fold change) >1 or< -1) between two different samples were calculated as the number of transcripts per million reads. RSEM (RNA-Seq by Expectation-Maximization) software (https://deweylab.github.io/RSEM/) was used to quantify gene abundances [26]. The data were analyzed through the free online platform of Majorbio Cloud Platform (http://www.majorbio.com).

### 2.13. Statistical Analysis

All data in this study are expressed as the mean ± standard error of the mean (SEM). Statistical analyses were performed using SPSS 12.0 (SPSS Inc., Chicago, IL, USA). Differences between groups were identified by analysis of variance and post hoc multiple comparisons were performed with the Dunnett's *t*-test. The significance of differences between two groups was determined using the independent samples *t*-test. A probability (*p*) value of ≤0.05 was considered statistically significant.

## 3. Results and Discussion

### 3.1. Calcium Acetate Aggravated A*β*-Induced Neurodegenerative Motor Impairments, while EGTA Inhibited Neurodegenerative Dysfunction and Extended the Life Span of A*β* Worms

Calcium is a ubiquitous second messenger that plays essential roles in numerous neurophysiological processes, including muscle contraction, nerve cell differentiation and proliferation, synaptic plasticity, and learning and memory. Many *in vitro* studies have reported that decreased calcium levels attenuated A*β* plaque formation, suggesting that control of calcium levels is a potential strategy for protection against AD.

**Figure 1:**
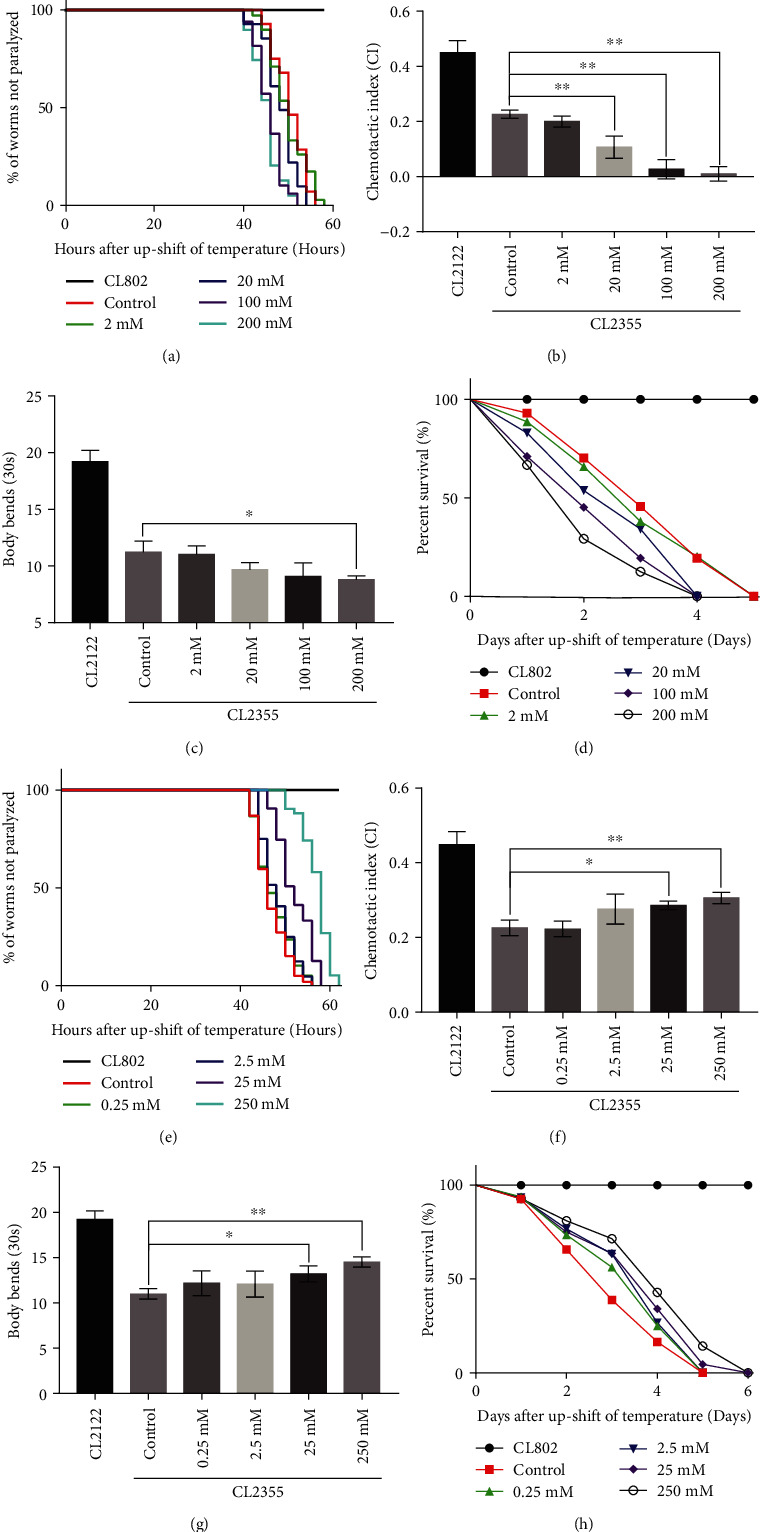
Calcium acetate aggravated A*β*-induced neurodegenerative motor impairments, while EGTA inhibited neurodegenerative dysfunction and extended the life span of A*β* worms. (a) Time course of A*β*-induced paralysis in CL4176 worms treated with/out calcium acetate. CL802 was the control strain. (b) CI values of calcium acetate -treated vs. untreated CL2355 worms. CL2122 was the control strain. (c) Body bends capacity of calcium acetate-treated vs. untreated CL2355 worms. CL2122 was the control strain. (d) Time course of lifespan of calcium acetate-treated vs. untreated CL4176 worms. CL802 was the control strain. (e) Time course of A*β*-induced paralysis in CL4176 worms treated with/out EGTA. (f) CI values of EGTA-treated vs. untreated CL2355 worms. (g) Body bends capacity of EGTA-treated vs. untreated CL2355 worms. (h) Time course of lifespan of EGTA-treated vs. untreated CL4176 worms. Error bars indicate SEM. (^∗^*p* < 0.05 vs. control, ^∗∗^*p* < 0.01 vs. control).

To investigate the effect of calcium on degeneration of motor neurons *in vivo*, CL4176 and CL2355 worms as models of A*β*-induced AD were exposed to calcium acetate to mimic high calcium content. Transgenic CL4176 worms were used as a model of AD expressing the human A*β*_1-42_ peptide with aggregates of the body wall muscle leading to progressive paralysis. In worms, calcium acetate (soluble calcium) was used to elevate the extracellular Ca^2+^ levels and facilitate calcium influx at the dose of 20 mM [27]. Calcium acetate at 20–200 mM significantly promoted the onset of paralysis in CL4176 worms (Figure 1(a)). Transgenic CL2355 worms with defective chemotaxis were used as an AD model expressing the human A*β*_1-42_ peptide with aggregates in neuronal cells to evaluate neuron-controlled behaviors (chemotaxis) and locomotion (body bends capacity). The CI was defined as the proportion of worms that were able to arrive at a particular location spotted with benzaldehyde as a chemoattractant. As shown in Figure 1(b), the CI values of groups treated with 20–200 mM calcium acetate were significantly reduced in a dose-dependent manner as compared to the control group. Similarly, decreased body bends capacity was also observed in the group treated with 200 mM calcium acetate (Figure 1(c)). The lifespan of CL4176 worm treated with 20–200 mM calcium acetate was significantly shortened (Figure 1(d)). Collectively, these results indicated that relatively higher calcium levels exacerbated A*β*-induced behavioral dysfunction and defective chemotaxis, resulting in a shortened lifespan.

Conversely, the calcium chelating agent EGTA was used to decrease calcium levels with the effective dose of 1 mM and 20 mM in the previous reports [27, 28]. Our study tested the EGTA dose (0.25-250 mM) in the *C. elegans* AD model, treatment of CL4176 worms with 25 and 250 mM EGTA significantly delayed the onset of paralysis, enhanced chemotaxis, increased the CI value and body bends capacity, and eventually extended the lifespan, compared to the control group (Figure 1(e)–1(h)). A decrease in extracellular calcium ameliorated A*β*-induced neurodegenerative motor impairments of AD worms, suggesting that decreased calcium inhibited the progression of AD by protecting against the toxicity of A*β* plaques.

### 3.2. Calcium Acetate Exacerbated A*β*-Induced Damage of Neurons and Synapses, while EGTA Protected against these Effects

Damage to acetylcholinergic neurons, which results in decreased availability of acetylcholine in the synaptic clefts, is the primary hallmark of the brain tissues of AD patients. However, the therapeutic effect of improving the acetylcholine content was less than ideal. The presence of A*β* peptides and tau proteins is reported to impair dopaminergic signaling [29]. To investigate the combined effect of A*β* and calcium on the major neurons, transgenic CL2355 worms with GFP-conjugated gamma-aminobutyric acid (GABA) or dopaminergic neurons were constructed, respectively. Worm phenotypes are detailed in Supplementary Table S1.

**Figure 2:**
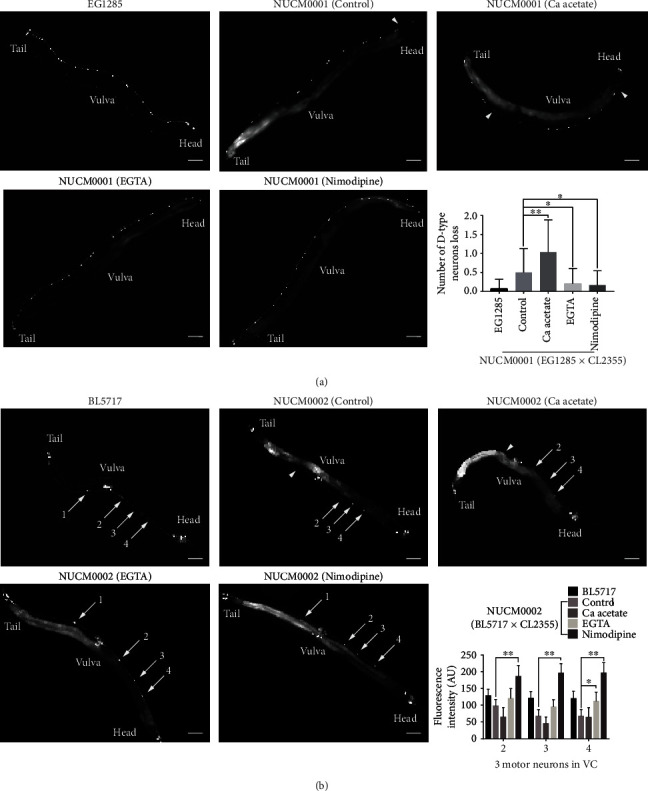
Calcium acetate aggravated impairment of GABAergic and motor neurons in the VNC, while EGTA and nimodipine postponed lesion development in A*β* worms. (a) Representative images of D-type GABAergic neurons in the VNCs of EG1285, NUCM0001, calcium acetate-treated NUCM0001, EGTA-treated NUCM0001, and nimodipine-treated NUCM0001 worms (scale bar, 50 *μ*m). Histogram showing the number of lost D-type GABAergic neurons in NUCM0001 worms treated with/out calcium-adjusting agents. Lost neurons are indicated by triangles. (b) Representative images of motor neurons in the VNCs of BL5717, NUCM0002, calcium acetate -treated NUCM0002, EGTA-treated NUCM0002, and nimodipine-treated NUCM0002 worms (scale bar, 50 *μ*m). Histogram of the fluorescence intensity of cell bodies of NUCM0002 worms treated with/out calcium-adjusting agents. Lost neurons are indicated with triangles. Motor neurons are indicated with arrows. Error bars indicate the SEM. (^∗^*p* < 0.05 vs. control, ^∗∗^*p* < 0.01 vs. control).

Locomotion of *C. elegans* is controlled by motor neurons and various neurotransmitters. The VNC of transgenic EG1285 worms had D-type GABAergic motor neurons similar to that of wild-type N2 worms. Also, the VNC of NUCM0001 (EG1285 × CL2355) worms had D-type GABAergic motor neurons similar to that of worms with A*β*-induced AD. Notably, the NUCM0001 worms had fewer D-type GABAergic motor neurons than EG1285 worms (Figure 2(a)).

As shown in Figure 2(b), the VNC of transgenic BL5717 worms had four motor neurons under control of the ida-1 promoter. The VNC of NUCM0002 (BL5717 × CL2355) worms had motor neurons similar to worms with A*β*-induced AD. As compared to BL5717 worms, the NUCM0002 progeny were missing the first motor neuron located in the area between the vulva and tail. The fluorescence intensity of neurons 2, 3, and 4 located from the vulva to pharyngeal pump was decreased in NUCM0002 worms, with significant decreases in neurons 3 and 4 as compared to the BL5717 worms.

**Figure 3:**
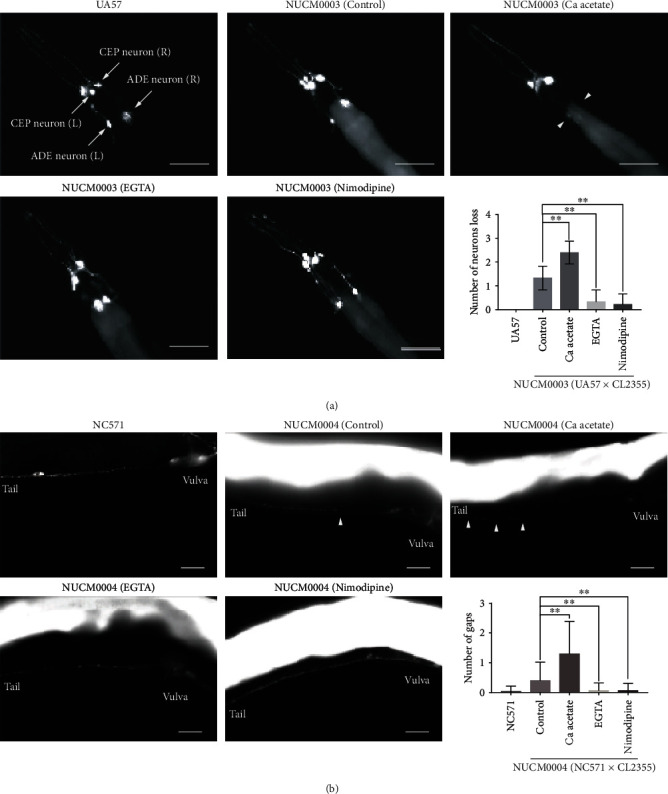
Calcium acetate aggravated impairment of dopaminergic neurons and synapses, while EGTA and nimodipine postponed the development of A*β* aggregates. (a) Representative images of dopaminergic neurons in UA57, NUCM0003, calcium acetate-treated NUCM0003, EGTA-treated NUCM0003, and nimodipine-treated NUCM0003 worms (scale bar, 50 *μ*m). Histogram of the numbers of lost dopaminergic neurons in NUCM0003 worms treated with/out calcium-adjusting agents. Lost neurons are indicated with triangles. Motor neurons are indicated with arrows. (b) Representative images of presynaptic densities in the VNCs of NC571, NUCM0004, calcium acetate-treated NUCM0004, EGTA-treated NUCM0004, and nimodipine-treated NUCM0004 worms (scale bar, 20 *μ*m). Histogram of the number of gaps in NUCM0004 worms treated with/out calcium-adjusting agents. Gaps are indicated with triangles. Error bars indicate the SEM. (^∗^*p* < 0.05 vs. control, ^∗∗^*p* < 0.01 vs. control).

As shown in Figure 3(a), the transgenic UA57 worms expressed GFP in four dopaminergic (two cephalic and two anterior deirid) neurons under control of the neuron-specific promoter of dat-1 in dat-1P::GFP worms. The NUCM0003 (UA57 × CL2355) worms had one or two fewer neurons. The brain tissues of the constructed transgenic worms (NUCM0001, NUCM0002, and NUCM0003) had abnormal A*β* aggregates that induced extensive impairment of the major (dopaminergic and GABAergic) neurons.

In order to visualize synapse morphology, transgenic NC571 worms were engineered to express GFP-tagged *synaptobrevin-1* (*SNB-1*) under control of the neuron-specific promoter of *unc-4*. SNB-1 is expressed in a subset of cholinergic motor neurons in the VNC. In transgenic NC571 worms, SNB-1:: GFP formed a regular pattern of puncta along the VNC. In addition, the VNC at the posterior gonadal arm was examined. As compared to strain NC571, imaging of the NUCM0004 (NC571 × CL2355) worms revealed a discontinuous punctate pattern and staining was generally more diffuse in presynaptic areas (Figure 3(b)). As SNB-1 is located in the presynaptic termini, these results confirm that A*β* aggregations damage synapses.

As shown in Figures 2 and 3, treatment of the four kinds of transgenic worms with 200 mM calcium acetate decreased the fluorescence intensity of motor neurons 2 and 3 in the VNC of NUCM0002 worms (Figure 2(b)), led to significant deterioration and loss of the GABAergic neurons of NUCM0001 worms and the dopaminergic neurons of NUCM0003 worms (Figure 2(a) and Figure 3(a)), and significantly exacerbated the limited accumulation of SNB-1 in the presynaptic termini of NUCM0004 worms (Figure 3(b)). These results indicated that an increase in calcium levels exacerbated A*β*-induced impairment of neurons and synapses.

In contrast, 250 mM EGTA reversed neuronal and synaptic deficiencies in the transgenic worms. As compared to the control group, EGTA significantly increased the fluorescence intensity of motor neuron 3, decreased the deterioration and loss of GABAergic and dopaminergic neurons, and suppressed the increased frequency of gaps (Figures 2 and 3).

These results showed that the combined actions of A*β* and calcium caused extensive damage to the nervous system, especially to GABAergic and dopaminergic neurons, rather than only acetylcholinergic neurons. Meanwhile, decreased calcium reduced A*β*-induced neurodegenerative dysfunction by ameliorating neuronal and synaptic deficiencies in *C*. *elegans* models of AD.

### 3.3. Nimodipine Rescued Neurodegenerative Motor Lesions, Ameliorated Neuronal and Synaptic Impairment, and Extended the Lifespan of A*β* Worms

**Figure 4:**
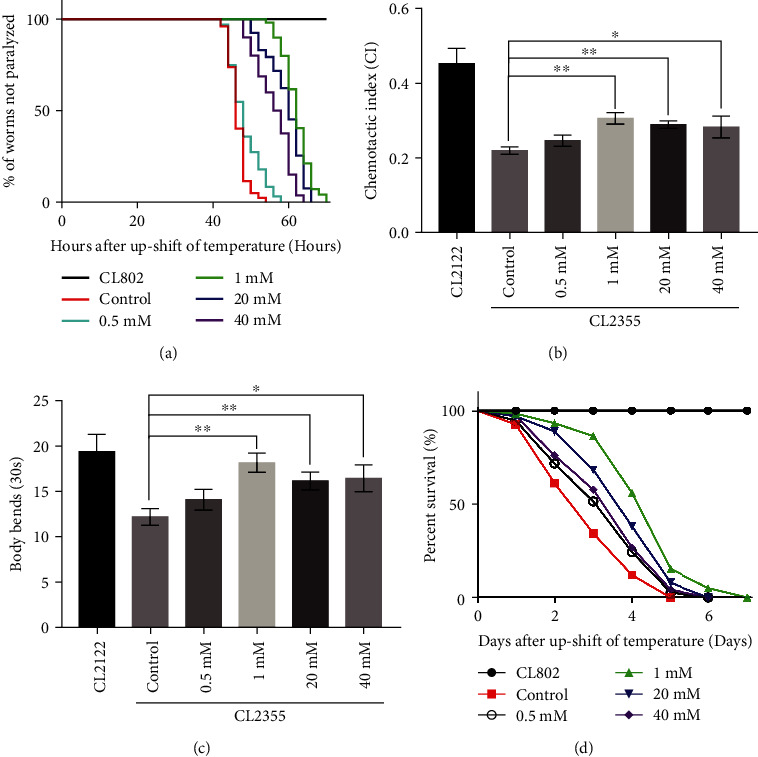
Nimodipine inhibited neuronal degeneration and extended the lifespan of A*β* worms. (a) Time course of A*β*-induced paralysis in CL4176 worms treated with/out nimodipine. CL802 was the control strain. (b) The CI of CL2355 worms treated with/out nimodipine. CL2122 was the control strain. (c) Body bends capacity of CL2355 worms treated with/out nimodipine. CL2122 was the control strain. (d) Time course of the lifespan of CL4176 worms treated with/out nimodipine. CL802 was the control strain. Error bars indicate the SEM. (^∗^*p* < 0.05 vs. control, ^∗∗^*p* < 0.01 vs. control).

Nimodipine, a voltage-gated calcium channel (VGCC) blocker, was used to test the effect of selective inhibition of calcium influx on A*β*-induced neuronal degeneration and dysfunction. Previous reports observed 20 *μ*M dose of nimodipine treated with the cortical cell culture significantly reduced the neuronal injury, treated with Schwann cells decreased cytotoxicity, treated with neurons facilitated neurite elongation [30–32]. However, this nimodipine dose could not significantly change chemotaxis and body bends in the *C. elegans* AD model (data not shown). We tested the influence of 0.5 mM-40 mM of nimodipine on the AD degeneration. As shown in Figure 4, treatment with 1, 20, and 40 mM of nimodipine significantly delayed the onset of paralysis, improved chemotaxis, the associative learning index, and body bends, and even extended lifespan, similar to the EGTA treatment. In the following experiments, 1 mM of nimodipine was used as effective dose to treat worms.

Treatment of transgenic worms expressing specific neurons in an A*β* background with 1 mM nimodipine had significantly ameliorated neuronal and synaptic damage, increased the fluorescence intensities of three motor neurons, reversed the loss of GABAergic and dopaminergic neurons, and restored accumulation of SNB-1 in the presynaptic termini (Figures 2 and 3). These results suggested that suppression of voltage-dependent calcium channels effectively inhibited A*β*-induced neurodegenerative dysfunction by ameliorating neuronal and synaptic deficiency in the AD models.

### 3.4. Calcium Acetate Intensified Aggregation of A*β* Plaques Induced by Oxidative Stress, while EGTA and Nimodipine Decreased the Aggregation of A*β* Plaques via Increased Antioxidative Stress in A*β* Worms

**Figure 5:**
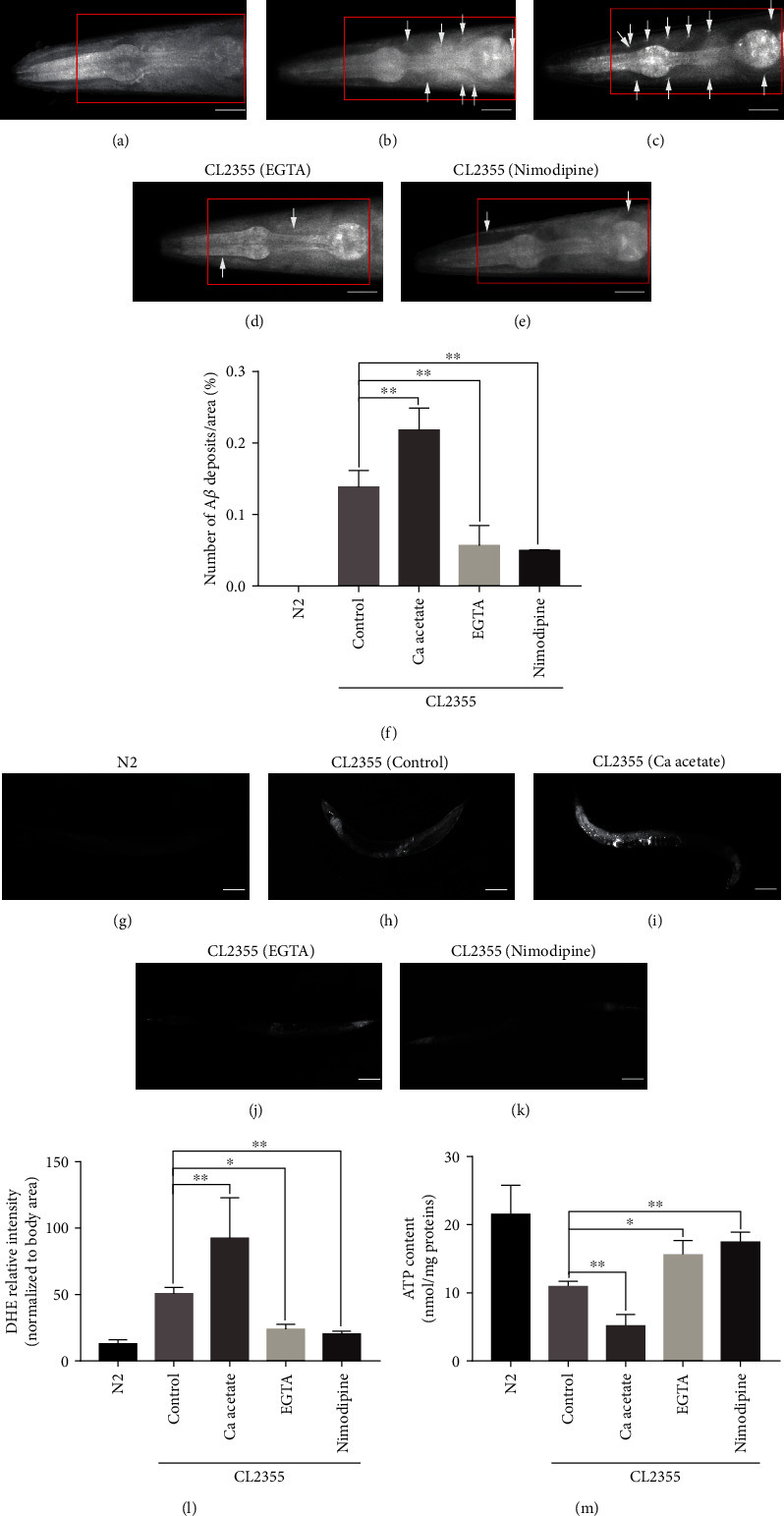
Calcium acetate aggravated A*β* aggregation by increasing oxidative stress in A*β* worm, while EGTA and nimodipine had opposite effects. (a‑e) Representative images of A*β* deposits in N2, CL2355, and CL2355 worms treated with calcium acetate, EGTA, and nimodipine (scale bar, 20 *μ*m). The area in the red rectangle was observed. Arrows indicate ThS staining. (f) Histogram of the mean number of A*β* deposits in the head region/anterior area of the worms. (g–k) Representative images of ROS content tested by the dihydroethidium (DHE) red fluorescence, in N2, CL2355, and CL2355 worms treated with calcium acetate, EGTA, and nimodipine (scale bar, 100 *μ*m). (l) Histogram of the mean ROS content. Error bars indicate the SEM. (m) Histogram of the mean ATP content in N2, CL2355, and CL2355 worms treated with calcium acetate, EGTA, and nimodipine. (^∗^*p* < 0.05 vs. control, ^∗∗^*p* < 0.01 vs. control).

To investigate the relationship between calcium and A*β* peptides in the progression of AD *in vivo*, the cellular localization of accumulated transgenic A*β*_1-42_ in response to treatment with different calcium-adjusting agents was examined by staining for ThS. Wild-type N2 worms served as a negative control due to negative staining for ThS in the head. In this experiment, the anterior areas of CL2355 worms administrated different calcium-adjusting agents were observed. As shown in Figure 5(a)–5(f), the anterior areas of CL2355 worms treated with calcium acetate showed extensive staining for ThS as compared to the control group and worms treated with EGTA or nimodipine. These results suggest that suppression of voltage-dependent calcium influx effectively inhibited A*β*-induced neuronal degeneration and dysfunction *in vivo* by suppressing aggregation of A*β* plaques.

Oxidative stress contributes to the development of AD. ATP synthesis is negatively correlated with the development and progression of AD, while ROS production is positively correlated. Therefore, the effect of calcium modifiers on the contents of ATP and ROS was investigated. Regarding to the ROS production, the CL2355 strain of *C. elegans* AD model presented significantly higher fluorescence, compared with the N2 wild-type. The ROS levels were increased in the calcium acetate group, but decreased in the EGTA and nimodipine groups (Figures 5(g)–5(l)). As shown in Figure 5(m), calcium acetate reduced ATP contents in CL2355 worms, while EGTA and nimodipine had opposite effects. These results suggested that suppression of voltage-dependent calcium influx improved ATP synthesis and reduced ROS production to protect against oxidation.

### 3.5. RNA Sequencing Revealed Up- and Down-regulated Genes between Nimodipine-Treated and Untreated CL4176 and CL2355 Worms

RNA sequencing was performed to identify genes associated with pathways potentially involved in nimodipine-mediated protection against A*β*-induced toxicity in nimodipine-treated and untreated CL4176 and CL2355 worms. The 46,904 gene expression values, reported in fragments per kilobase of transcripts per million mapped fragments (FPKM), from all worm groups are listed in Supplementary Table S3. Among these, 14,491 genes with an expression value >0 are listed in Supplementary Table S4.

In total, 2,306 genes were upregulated and 618 were downregulated in nimodipine-treated vs. untreated CL4176 worms (Supplementary Table S5), while 3,820 genes were upregulated and 1,352 were downregulated in nimodipine-treated vs. untreated CL2355 worms (Supplementary Table S6).

### 3.6. Nimodipine Induced Significant Changes to the Transcript Levels of Genes Associated with Calcium Signaling, Neurotransmitters, and Antioxidative Stress Pathways in CL2355 Worms

The results of RNA sequencing revealed that the transcript levels of nine genes associated with calcium signaling were significantly upregulated in CL2355 worms treated with nimodipine as compared to untreated worms. As shown in Table 1, they are ryanodine receptor gene (*unc-68*), calcium binding protein (*cbn-1*), calcium-transporting ATPase (*mca-2*), calcium homeostasis modulator protein (*clhm-1)*, Na/Ca^2+^ exchangers (*ncx-1*), neuronal calcium sensor family (*ncs-4*), small conductance calcium-activated potassium channel-like protein 3 (*kcnl-3*), voltage-gated ion channel alpha-1 subunit (*nca-2*), and calcineurin-interacting protein 2 (*cnp-2*). Also, nimodipine-induced suppression of calcium influx through VGCCs resulted in upregulated expression of acetylcholinergic receptor genes (*acr-8*, *acr-2*, *unc-29*, *des-2*, d*eg-3*, *acr-3*, *acc-4*, *acr-9*, *gar-2*, and *lev-1*), GABAergic receptor genes (*unc-49* and *ggr-2*), and a dopaminergic receptor gene (*dop-1*). These results suggested that suppression of selective VGCCs improved the functions all three types of neurotransmitters.

Because nimodipine is also reported to increase resistance to oxidative stress in brain injury [31, 32], the transcript levels of genes relevant to oxidative stress resistance were considered. As described in Table 1, the transcript levels of genes related to the *C*. *elegans* ortholog of human hematopoietic prostaglandin D synthase (HPGDS), including *gst-3*, *gst-8*, *gst-9*, *gst-12*, *gst-20*, *gst-21*, *gst-24*, *gst-30*, *gst-31*, *gst-35*, and *gst-38,* were significantly upregulated, as were genes associated with glutathione S-transferase (GST) activity (*gst-10* and *gst-25*). However, genes associated with GST activity (*gstk-1*) and glutathione peroxidase activity (*pgx-7*) were significantly downregulated in nimodipine-treated CL2355 worms as compared to untreated controls. HPGDS (GST Sigma) catalyzes glutathione conjugation to molecules and plays a role in prostaglandin D2 biosynthesis, associated with the inflammatory response [33, 34]. GST is a large family generally involved in detoxification and removal of oxidative stress. One family member of GSTP (*gst-10* homolog) acts as a sensor of intracellular redox potential associated with neuritogenesis, and interacts with JNK1 to inhibit oxidative stress-induced apoptosis [35, 36]. These results suggested that nimodipine upregulated the transcript levels of numerous genes associated with calcium signaling, neuronal receptors, and antioxidative enzymes (i.e., HPGDS and GSTs).

**Figure 6:**
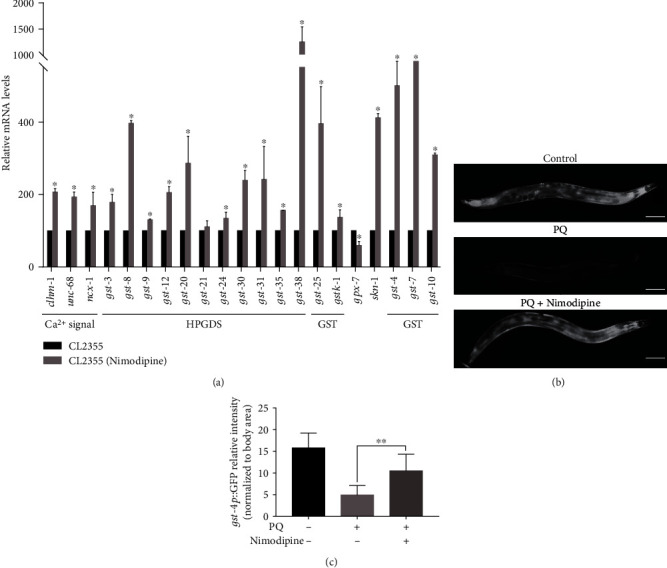
Nimodipine upregulated the transcript levels of genes encoding antioxidative enzymes (HPGDS and GST). (a) Relative mRNA levels of genes relevant to antioxidative stress in CL2355 worms treated with/out nimodipine. (b) Representative images of *gst-4_P_*::GFP expression in CL2166 and CL2166 worms treated with PQ, CL2166, and nimodipine (scale bar, 100 *μ*m). (c) Histogram of *gst-4_P_*::GFP relative fluorescence intensity/body area of the worm.

In *C. elegans*, the major ROS detoxification mechanisms are initiated by the transcription factor SKiNhead-1 (SKN-1). SKN-1 plays key roles in increasing oxidative stress resistance and extending lifespan by scavenging free radicals. In response to oxidative stress, SKN-1 promote transcription of genes associated with phase II detoxification, such as *gst-4*, *gst-7*, and *gst-10*, possibly via interactions between SKN-1/Nrf nuclear factor and the antioxidant responsive element of the *GST* promoter region [35, 37, 38]. Therefore, the transcript levels of orthologous genes of HPGDS and GST, and those related to oxidative stress resistance (*skn-1*) were detected by qRT-PCR. As shown in Figure 6(a), as compared to the control group, nimodipine administration significantly up-regulated the transcript levels of *gst-3*, *gst-8*, *gst-9*, *gst-12*, *gst-20*, *gst-24*, *gst-25, gst-30*, *gst-31*, *gst-35*, *gst-38*, and *gst-10*, and significantly downregulated expression of *gpx-7*, consistent with the results of RNA sequencing. Although changes to the transcript levels of *skn-1*, *gst-4*, and *gst-7* were not significant according to the RNA sequencing results, qRT-PCR analysis determined that all were significantly upregulated. Notably, *gst-4*, which is downstream of *skn-1*, is commonly used as an important antioxidative indicator. Nimodipine treatment increased GFP expression in *gst-4_P_::GFP* transgenic CL2166 worms with PQ-induced oxidative stress (Figures 6(b) and 6(c)). These results further verified that nimodipine increased oxidative stress resistance by promoting expression of HPGDS and GSTs.

### 3.7. Suppression of Selective VGCC Enhanced Oxidative Stress Resistance via Regulation of HPGDS and GST Activities in CL2355 A*β* Worms

Three of the nine upregulated genes related to calcium signaling (i.e., *clhm-1*, *unc-68*, and *ncx-1*) were selected to evaluate influences on oxidative stress resistance in nimodipine-treated CL2355 worms. Of these, *clhm-1* codes for a calcium-permeable ion channel regulated by voltage and extracellular calcium. As human homologs, members of the calcium homeostasis modulator (CALHM) family form calcium-regulated ATP-release channels involved in neuronal functions [39]. In *C. elegans*, heterogeneous expression of *clhm-1* in neurons displays toxic degeneration, while the muscle-specific expression requires for coordinated locomotion [40]. Thus, the role of *clhm-1* in AD neuronal degeneration needs to be further clarified.

*Unc-68* encodes for the ryanodine receptor (RyR), which is involved in calcium release from the endoplasmic reticulum and required for regeneration in response to neuronal damage [41]. In addition, *unc-68* is also expressed in body-wall muscle cells that are used for locomotion. Worms with mutated *unc-68* exhibit incomplete flaccid paralysis, yet the muscle ultrastructure is normal [42, 43]. As human homologs, RyR family proteins form calcium release channels in the sarcoplasmic reticulum in striated muscle that regulate excitation-contraction coupling in cardiac muscle [44]. Consistent with the RNA sequencing data, it suggests *unc-68* upregulation in the nimodipine-treated worms could be necessary for rescue of neuronal degeneration.

*Ucx-1 enc*odes a sodium-calcium exchanger located in the axons, postsynapses, and sarcolemma that transports sodium and calcium ions across the plasma membrane [45]. As a human ortholog, solute carrier family 8 member A has been implicated in AD and pulmonary hypertension. An alternative splicing variant (AD-CBD2) of ncx-1 is highly expressed in neurons and encodes the acidic segment of calcium-binding site 2, which enables binding with calcium to attenuate inactivation NCX1 caused by high sodium levels [46]. Although ncx-1 function have not well studied yet in worms, our findings suggest its high expression associated with calcium homeostasis and required for alleviating neuronal damage.

**Figure 7:**
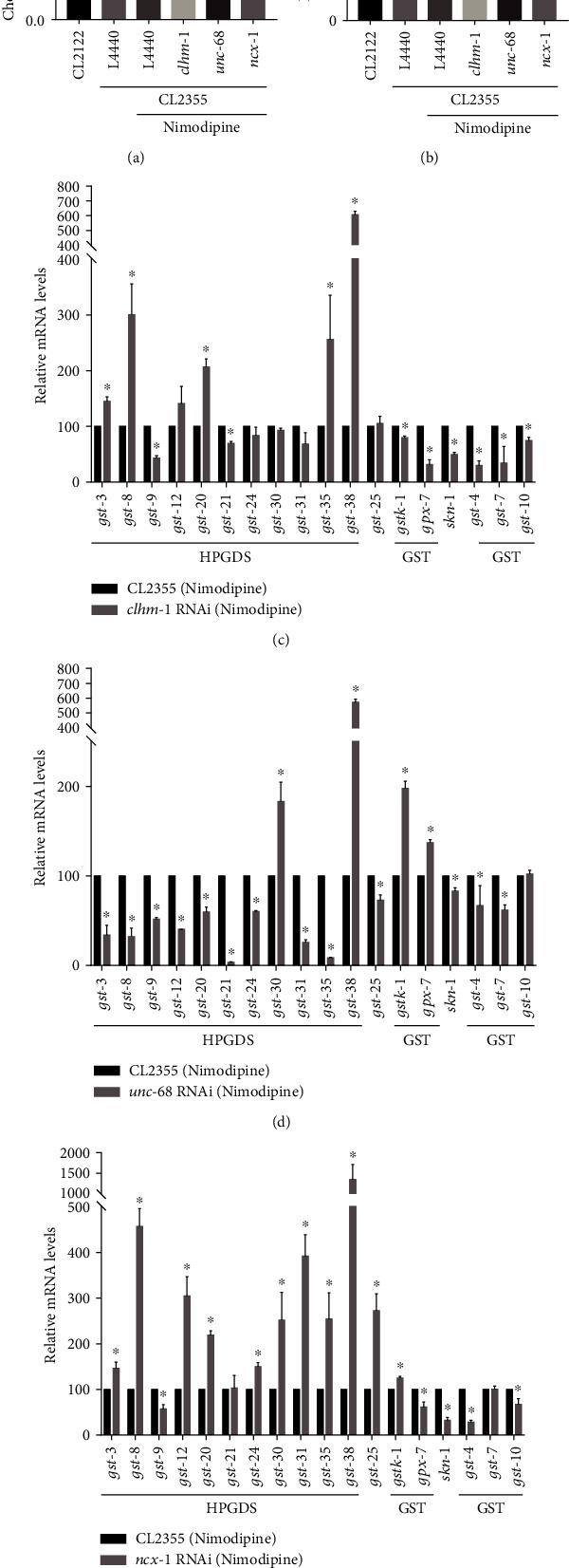
Suppression of selective VGCCs enhanced oxidative stress resistance through regulation of HPGDS and GST activities in CL2355 A*β* worms. (a) Activity of *clhm-1*, *unc-68*, or *ncx-1* in regulating the effect of nimodipine on the CI value. L4440 is an empty vector. (b) Activity of *clhm-1*, *unc-68*, or *ncx-1* in regulating the effect of nimodipine on body bending capacity. L4440 is an empty vector. (c) Relative mRNA levels of genes relevant to antioxidative stress in CL2355 and CL2355 *clhm-1* (RNAi) worms treated with nimodipine (d) Relative mRNA levels of genes relevant to antioxidative stress in CL2355 and CL2355 *unc-68* (RNAi) worms treated with nimodipine. (e) Relative mRNA levels of genes relevant to antioxidative stress in CL2355 and CL2355 *ncx-1* (RNAi) worms treated with nimodipine. The treatment concentration of nimodipine was 1 mM. Error bars indicate the SEM. (^∗^*p* < 0.05 vs. control, ^∗∗^*p* < 0.01 vs. control).

As indicated by the qRT-PCR results presented in Figure 6(a), *clhm-1, unc-68*, and *ncx-1* were all upregulated in the nimodipine groups as compared to the control group, in agreement with the results of RNA sequencing. Second, RNAi of these genes was performed to assess nimodipine-induced changes to motor and learning capacities. The qRT-PCR assay was used to determine the RNAi efficiency, whose results showed persistent RNAi on the second generation with significant decrease of the target transcript levels (Supplementary Figure S1). As shown in Figures 7(a) and 7(b), both the CI and body bends decreased in nimodipine-treated CL2355 worms with RNAi of *clhm-1*, *unc-68*, and *ncx-1*. These results suggested that nimodipine increased the activities of *clhm-1*, *unc-68*, and *ncx-1*, as a protective role against A*β*-induced toxicity.

Lastly, the transcript levels of orthologous genes of HPGDS and GST, and *skn-1* were compared separately in nimodipine-exposed CL2355 worms with RNAi of *clhm-1*, *unc-68*, or *ncx-1* (Figures 7(c), 7(d), and 7(e), respectively). In nimodipine-treated *clhm-1* (RNAi) CL2355 worms, the transcript levels of *skn-1*, *gst-4*, *gst-7*, and *gst-10* were significantly downregulated as compared to CL2355 worms only exposed to nimodipine (Figure 7(c)). Also, *skn-1*, g*st-4*, and *gst-7* were downregulated in *unc-68* (RNAi) CL2355 worms treated with nimodipine (Figure 7(d)), while *skn-1, gst-4*, and *gst-10* were downregulated in *ncx-1* (RNAi) CL2355 worms after nimodipine treatment (Figure 7(e)). Hence, *skn-1* was obviously regulated by *clhm-1*, *unc-68*, or *ncx-1*, as well as downstream genes encoding GSTs. These results indicated that nimodipine could increase oxidative stress resistance by upregulating expression of *clhm-1*, *unc-68*, and *ncx-1* to strengthen *skn-1* and then GSTs.

In addition, nimodipine treatment of RNAi CL2355 worms induced significant changes to the transcript levels of orthologous genes of *HPGDS*, including *gst-3*, *gst-8*, *gst-9*, *gst-20*, *gst-35*, and *gst-38*, as compared to CL2355 worms only exposed to nimodipine (Figures 7(c)–7(e)), indicating that HPGDS was regulated by *clhm-1*, *unc-68*, and *ncx-1.* These genes are predicted to activate GSTs in *C. elegans* (https://www.wormbase.org). The tertiary structure of HPGDS, which is a cytoplasmic GST, has an N-terminal Trx-like domain. Trx-like homologs can act as antioxidants and participate in intracellular signaling to protect cells against peroxides [47]. Recent studies discovered that HPGDS specifically catalyzes the isomerization of PGH2 to PGD2, which results in activation of Nrf2 in bone marrow-derived macrophages [48]. In this study, the transcript levels of some orthologous genes of HPGDS and *skn-1* were commonly influenced by RNAi of *clhm-1*, *unc-68*, and *ncx-1* (Figures 7(c)–7(e)). These results indicated that HPGDS likely acted as a signal messenger to increase oxidative stress resistance by upregulating *skn-1* expression to strengthen GST activity in nimodipine-induced antioxidative stress.

## 4. Conclusions

**Figure 8:**
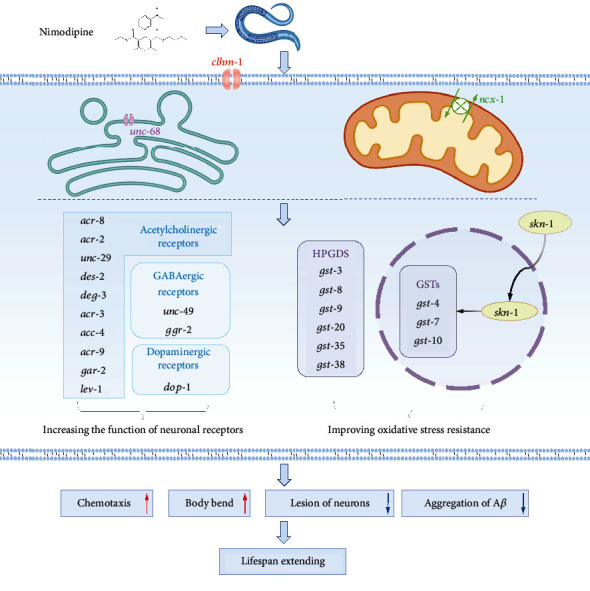
Graphic illustrating nimodipine induced regulation of calcium homeostasis and oxidative resistance against AD degeneration. Nimodipine suppression on the voltage-gated channels (VGCCs) could induce other calcium-signal complements, including Ca^2+^-permeable ion channel *chlm-1* and ER Ca^2+^ release related ryanodine receptor *unc-68*, sodium-calcium exchanger *ncx-1*, all these proteins essential for neuron development. Calcium reequilibrium facilitates *skn-1-gst/HPGDS*-mediated oxidative resistance. Meanwhile, nimodipine increases the transcript levels of acetylcholinergic, GABAergic, and dopaminergic receptor genes. Combinational effects of the enhancing neuronal system function and oxidative resistance relieve A*β*-induced neuronal degeneration and dysfunction, thus extend lifespan.

Calcium acetate exacerbated A*β*_1–42_ aggregation through oxidative stress, then aggravated neuronal degeneration and dysfunction, which shortened the lifespan. In contrast, nimodipine-induced suppression of calcium influx significantly induced other calcium channels of the plasma membrane (*clhm-1*), activated intercellular ER calcium channel (*unc-68*) and sodium-calcium exchanger channels (*ncx-1*), and further upregulated downstream genes, such as orthologous genes of HPGDS and *skn-1*, thereby strengthening GST activity. These results indicated that suppression of selective VGCCs could enhance oxidative stress resistance via upregulation of *clhm-1*, *unc-68*, and *ncx-1* to strengthen HPGDS and *skn-1*, and then GST, further inhibiting A*β*_1–42_ aggregation, to ameliorate neuronal degeneration and dysfunction in AD worms (the graphical mechanism illustrated in Figure 8). These findings reveal the underlying regulatory mechanisms of calcium-mediated oxidative resistance against the AD degeneration, potentially provide multiple drug targets, and could be applied to devise new strategies for the AD treatment.

## Figures and Tables

**Table 1:** Differentially expressed genes relevant to calcium signaling, neurotransmitters, and oxidative stress in nimodipine-treated vs. untreated A*β* worms.

Gene symbol	Log2FC	*p* value	Regulated type	Gene description
Nimodipine-treated CL2355 vs. CL2355				
*Unc-68*	1.09	1.40E-02	Up	Calcium-induced calcium release activity
*Cbn-1*	2.33	1.26E-05	Up	Calcium binding protein homolog
*Mca-2*	1.35	1.64E-08	Up	Calcium-transporting ATPase
*Clhm-1*	1.58	1.34E-04	Up	Calcium homeostasis modulator protein
*Ncx-1*	2.16	3.49E-06	Up	Na/ca exchangers
*Ncs-4*	1.68	1.95E-03	Up	Neuronal calcium sensor family
*Kcnl-3*	1.08	4.90E-02	Up	Calcium-activated potassium channel-like protein 3
*Nca-2*	1.09	1.59E-02	Up	Voltage-gated ion channel alpha-1 subunit
*Cnp-2*	3.02	1.34E-21	Up	Calcineurin-interacting protein 2
*Acr-8*	2.14	6.34E-04	Up	Acetylcholine receptor
*Acr-2*	1.36	1.36E-02	Up	Acetylcholine receptor subunit beta-type
*Unc-29*	1.37	7.91E-03	Up	Acetylcholine receptor subunit beta-type
*Des-2*	1.32	6.85E-04	Up	Acetylcholine receptor subunit alpha-type
*Deg-3*	1.19	2.58E-03	Up	Acetylcholine receptor subunit alpha-type
*Acr-3*	1.17	1.26E-02	Up	Acetylcholine receptor subunit beta-type
*Acc-4*	1.25	1.51E-02	Up	Acetylcholine-gated ion channel
*Acr-9*	1.65	3.23E-02	Up	Acetylcholine receptor
*Gar-2*	1.19	1.62E-05	Up	Muscarinic acetylcholine receptor
*Lev-1*	1.28	1.29E-04	Up	Acetylcholine receptor subunit beta-type
*Unc-49*	1.47	7.75E-06	Up	Ionotropic GABA receptor subunit UNC-49
*Ggr-2*	1.35	4.12E-02	Up	GABA/glycine receptor family
*Dop-1*	1.01	4.65E-02	Up	Dopamine receptor
*Gst-8*	3.01	1.26E-02	Up	An ortholog of human HPGDS
*Gst-9*	3.11	1.38E-04	Up	An ortholog of human HPGDS
*Gst-10*	1.71	1.72E-03	Up	An ortholog of human GSTP1 (GST pi 1)
*Gst-31*	1.87	2.17E-02	Up	An ortholog of human HPGDS
*Gst-38*	2.89	1.16E-02	Up	An ortholog of human HPGDS
*Gst-3*	1.92	1.07E-04	Up	An ortholog of human HPGDS
*Gst-12*	1.09	3.78E-03	Up	An ortholog of human HPGDS
*Gst-20*	1.01	2.71E-04	Up	An ortholog of human HPGDS
*Gst-21*	4.02	6.05E-05	Up	An ortholog of human HPGDS
*Gst-24*	1.23	3.70E-03	Up	An ortholog of human HPGDS
*Gst-30*	1.84	1.32E-02	Up	An ortholog of human HPGDS
*Gst-35*	3.91	1.22E-03	Up	An ortholog of human HPGDS
*Gst-25*	3.04	1.21E-02	Up	An ortholog of GST
*Gstk-1*	-1.00	4.12E-06	Down	An ortholog of human GSTK1 (GST kappa 1)
*Gpx-7*	-1.51	2.20E-08	Down	Glutathione peroxidase
Nimodipine-treated CL4176 vs CL4176				
*Gst-6*	1.69	7.55E-15	Up	An ortholog of human HPGDS
*Gst-19*	6.09	5.52E-04	Up	An ortholog of human HPGDS
*Gst-36*	1.01	3.62E-05	Up	An ortholog of human HPGDS
*Gst-44*	1.86	3.76E-07	Up	An ortholog of human GSTO1 and GSTO2
*Gstk-1*	1.37	4.11E-03	Up	An ortholog of human GSTK1 (GST kappa 1)
*Gpx-7*	1.96	2.67E-08	Up	Glutathione peroxidase
*Gst-9*	-1.85	4.07E-03	Down	An ortholog of human HPGDS
*Gst-38*	-1.44	3.46E-04	Down	An ortholog of human HPGDS
*Gst-35*	-1.21	4.30E-02	Down	An ortholog of human HPGDS
*Gst-15*	-1.09	3.63E-05	Down	An ortholog of human HPGDS
*Gst-34*	-3.16	1.85E-03	Down	An ortholog of human HPGDS
*Gst-22*	-2.10	1.82E-11	Down	An ortholog of GST

Note: Log2FC means Log2(FPKM_Nimodipine-treated (CL2355 or CL4176)_/FPKM_(CL2355 or CL4176)_, HPGDS means hematopoietic prostaglandin D synthase, GST means glutathione S-transferase, GSTO means GST omega.

## Data Availability

The data used to support the findings of this study are included within the article and the supplementary information files.

## Supplementary Materials

Supplementary 1 Supplementary Figure S1. The RNAi efficiency assessed by qRT-PCR. RNAi of *clhm-1*, *unc-68*, and *ncx-1* treated with CL2355 worms, starting from the first-generation larva to the second-generation adult. The qRT-PCR results showed persistent RNAi on the second generation with significant decrease of the target transcript levels.

Supplementary 2 Supplementary Table 1. Description of the worm strains used in this study.

Supplementary 3 Supplementary Table 2. Primers for RNAi and qRT-PCR analyses.

Supplementary 4 Supplementary Table 3. Table of all gene expression values (FPKM) in different worm groups. The columns list the Wormbase ID, Gene Symbol, CL4176 FPKM, Nimodipine-treated CL4176 FPKM, CL2355 FPKM, and nimodipine-treated CL2355 FPKM. The other columns show the FPKM values of genes in each worm group.

Supplementary 5 Supplementary Table 4. Table of gene expression values (FPKM) above zero in different worm groups. The columns in the table list the Wormbase ID, Gene Symbol, CL4176 FPKM, Nimodipine-treated CL4176 FPKM, CL2355 FPKM, and Nimodipine-treated CL2355 FPKM. The other columns show the FPKM values of genes in each worm groups.

Supplementary 6 Supplementary Table 5. Significant differentially expressed genes between nimodipine-treated and untreated CL4176 worms. The columns in the table list the Wormbase ID, Gene Symbol, Fold_Change (nimodipine-treated CL4176/CL4176), log2(fold change), significant, *p* value, and Type. CL4176 FPKM, Nimodipine-exposed CL4176 FPKM, Wormbase ID column is the ID based on Wormbase database. CL4176 FPKM and Nimodipine-treated CL4176 FPKM are the FPKM values of gene in sample CL4176 and Nimodipine-exposed CL4176. Type column mark the up- or downregulated genes.

Supplementary 7 Supplementary Table 6. Significant differentially expressed genes between nimodipine-treated and untreated CL2355 worms. The columns in the table list the Wormbase ID, Gene Symbol, Fold Change (nimodipine-treated CL2355/CL2355), log2(fold change), significant, *p* value, and Type. CL2355 FPKM, Nimodipine-exposed CL2355 FPKM, Wormbase ID column is the ID based on Wormbase database. CL2355 FPKM and Nimodipine-treated CL2355 FPKM are the FPKM values of genes identified in nimodipine-treated and untreated CL2355 worms. The up- and downregulated genes are indicated.
