# Supplementary figures and images for: Suppression of Selective Voltage-Gated Calcium Channels Alleviates Neuronal Degeneration and Dysfunction through Glutathione S-Transferase-Mediated Oxidative Stress Resistance in a Caenorhabditis elegans Model of Alzheimer's Disease
Source: Oxid Med Cell Longev. 2022 Nov 30;2022:8287633. doi: 10.1155/2022/8287633 (PMC9806690; doi:10.1155/2022/8287633)

**Supplementary Figure S1**

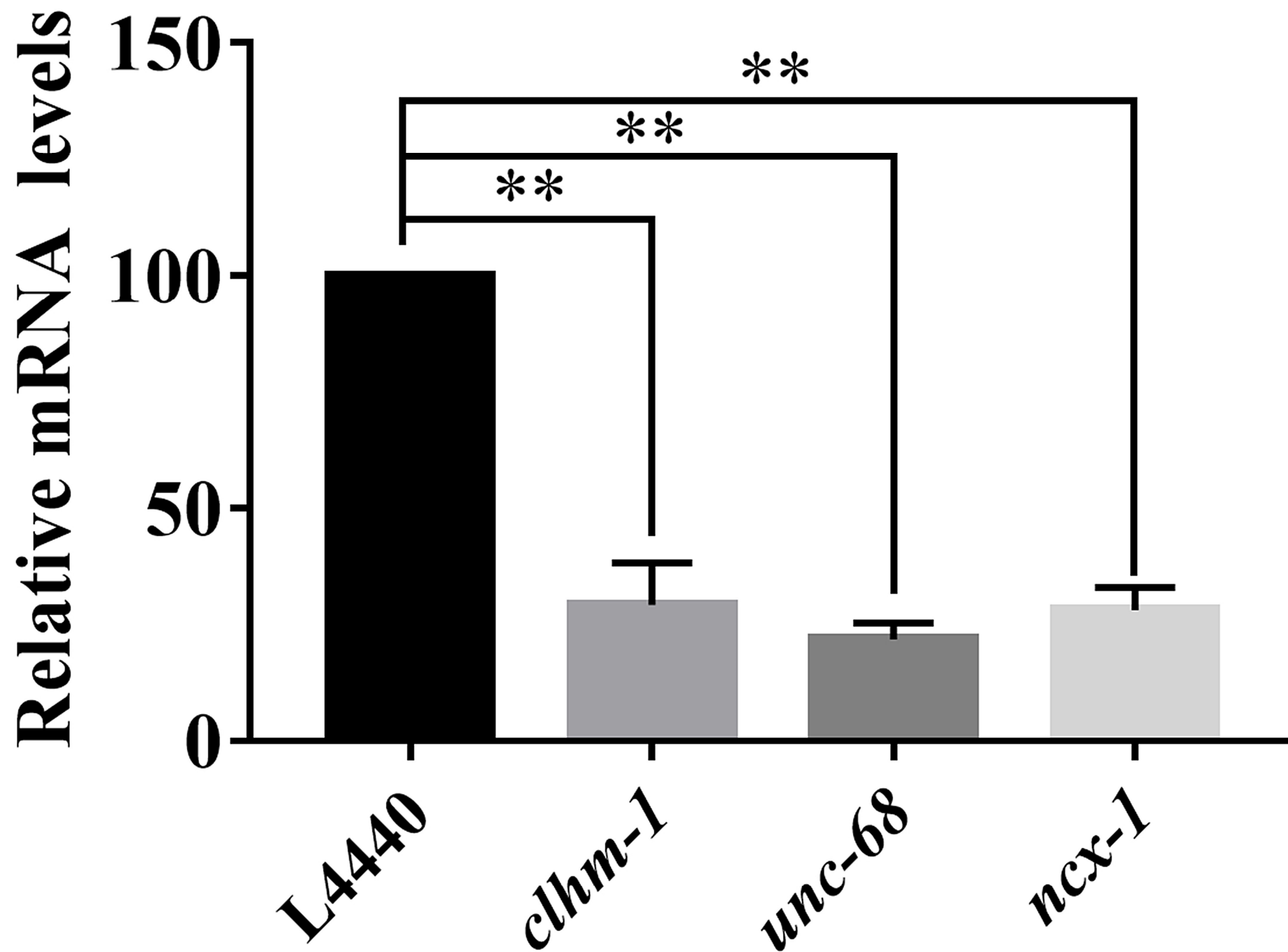

Supplement: Supplementary 1 — Supplementary Figure S1. The RNAi efficiency assessed by qRT-PCR. RNAi of clhm-1, unc-68, and ncx-1 treated with CL2355 worms, starting from the first-generation larva to the second-generation adult. The qRT-PCR results showed persistent RNAi on the second generation with significant decrease of the target transcript levels. [file 8287633.f1.pdf]
