# Supplementary material for: Suppression of Selective Voltage-Gated Calcium Channels Alleviates Neuronal Degeneration and Dysfunction through Glutathione S-Transferase-Mediated Oxidative Stress Resistance in a Caenorhabditis elegans Model of Alzheimer's Disease
Source: Oxid Med Cell Longev. 2022 Nov 30;2022:8287633. doi: 10.1155/2022/8287633 (PMC9806690; doi:10.1155/2022/8287633)
Supplement: Supplementary 2 — Supplementary Table 1. Description of the worm strains used in this study. [file 8287633.f2.pdf]

Supplementary Table S1

Strain	Transgene	Phenotype
N2	-	Wild type
CB4088	*him-5*(e1490)	Male worms
CL4176	*myo-3P*::Aβ_{1-42}:: + rol-6(su1006)	Rapid paralysis at 23℃ [1]
CL2355	*snb-1*::Aβ_{1-42} + *mtl-2:*:GFP	Deficits in chemotaxis, associative learning, and thrashing in liquid [2, 3]
CL2122	unc54(vector) + *mtl-2*::GFP	Phenotype apparently WT. Control strain for CL2355 in the study [4]
CL802	*smg-1*(cc546) + rol-6(su1006)	Control strain for CL4176 [5]
CL2166	*gst-4P*::GFP	Expression of GFP by improve *gst-4* function [6]
EG1285	*unc-47P*::GFP	Expression of GFP in all GABAergic neurons [7]
BL5717	*ida-1P*::GFP	Expression of GFP in 4 motor neurons in ventral nerve cord [8]
UA57	*dat-1P*::GFP+ *dat-1P*::CAT-2	GFP expression in cephalic (CEP), and anterior deirid (ADE) dopaminergic neurons [9]
NC571	*unc-4P*::*snb-1*::GFP	Punctate GFP expression observed in ventral nerve cord, VC4 and VC5 [8]
NUCM0001	*snb-1*::Aβ_{1-42} + *mtl-2*::GFP + *unc-47P*::GFP	Expression of GFP in all GABAergic neurons of Aβ-induced AD nematodes in the study
NUCM0002	*snb-1*::Aβ_{1-42} + *mtl-2*::GFP + *ida-1P*::GFP	Expression of GFP in 4 motor neurons in ventral nerve cord, of Aβ-induced AD nematodes in the study
NUCM0003	*snb-1*::Aβ_{1-42} + *mtl-2*::GFP + *dat-1P*::GFP+ *dat-1P*::CAT-2	GFP expression in CEP, and ADE neurons of Aβ-induced AD nematodes in the study
NUCM0004	*snb-1*::Aβ_{1-42} + *mtl-2*::GFP + *unc-4P*::*snb-1*::GFP	Punctate GFP expression observed in ventral nerve cord,, VC4 and VC5 of Aβ-induced AD nematodes in the study

Note: NUCM0001, NUCM0002, NUCM0003, NUCM0004 are generated by crossing in the study, other strains are obtained from the *Caenorhabditis* Genetics Center (https://cgc.umn.edu/).

References:

[1] Link CD, “Expression of human beta-amyloid peptide in transgenic Caenorhabditis elegans,” Proc Natl Acad Sci U S A, vol. 92, no. 20, pp. 9368-72, 1995.

[2] Wu Y, Wu Z, Butko P et al, “Amyloid-beta-induced pathological behaviors are suppressed by Ginkgo biloba extract EGb 761 and ginkgolides in transgenic Caenorhabditis elegans,” J Neurosci, vol. 26, no. 50, pp. 13102-13, 2006.

[3] Dosanjh LE, Brown MK, Rao G, Link CD, Luo Y, “Behavioral phenotyping of a transgenic Caenorhabditis elegans expressing neuronal amyloid-beta,” J Alzheimers Dis, vol. 19, no. 2, pp. 681-90, 2010.

[4] Chen XY, Liao DC, Sun ML, Cui XH, Wang HB, “Essential Oil of Acorus tatarinowii Schott Ameliorates Aβ-Induced Toxicity in Caenorhabditis elegans through an Autophagy Pathway,” Oxid Med Cell Longev, vol. 2020, p. 3515609, 2020 Dec 22.

[5] Fonte V, Dostal V, Roberts CM, "A glycine zipper motif mediates the formation of toxic β-amyloid oligomers in vitro and in vivo," Mol Neurodegener, vol. 6, no. 1, pp. 61, 2011.
[6] Wang X, Li H, Liu Y et al, "Velvet antler methanol extracts (MEs) protects against oxidative stress in Caenorhabditis elegans by SKN-1," Biomed Pharmacother, pp. 121:109668, 2020.
[7] Ruan Q, Qiao Y, Zhao Y et al, "Beneficial effects of Glycyrrhizae radix extract in preventing oxidative damage and extending the lifespan of Caenorhabditis elegans," J Ethnopharmacol, vol. 177, pp. 101-10, 2016.
[8] Estevez AO, Mueller CL, Morgan KL et al, "Selenium induces cholinergic motor neuron degeneration in Caenorhabditis elegans," Neurotoxicology, vol 33, no. 5, pp. 1021-32, 2012.
[9] Wang C, Saar V, Leung KL, Chen L, Wong G, "Human amyloid β peptide and tau co-expression impairs behavior and causes specific gene expression changes in Caenorhabditis elegans," Neurobiol Dis, vol. 109, no. Pt A, pp. 88-101, 2018.
