# Supplementary material for: Suppression of Selective Voltage-Gated Calcium Channels Alleviates Neuronal Degeneration and Dysfunction through Glutathione S-Transferase-Mediated Oxidative Stress Resistance in a Caenorhabditis elegans Model of Alzheimer's Disease
Source: Oxid Med Cell Longev. 2022 Nov 30;2022:8287633. doi: 10.1155/2022/8287633 (PMC9806690; doi:10.1155/2022/8287633)
Supplement: Supplementary 3 — Supplementary Table 2. Primers for RNAi and qRT-PCR analyses. [file 8287633.f3.pdf]

Supplementary Table S2

RNAi primer

Gene name	Forward	Reverse
clhm-1	ATACTGCAGCGTGCGAACAAGGG	CATGGTACCGGCTGTGGAAATGG
unc-68	ATAGGTACCCACTTGGCAACCAT	AATCTGCAGTGGTGAATGCTGCT
ncx-1	ATACTGCAGGTGGAATCAGGGCA	CTCGGTACCGTCATGTGGCTCAT

qRT-PCR primer

Gene name	Forward	Reverse
human-Aβ	GATGCAGAATTCCGACATGA	TCGCTATGACAACACCGCCCA
act-3	ATCCGTAAGGACTTGTACGCCAAC	GGGCGATGATCTTGATCTTCATGG
gst-3	ATTTTAATGCACGCGGACTT	CCTCTGCAGTTTGACCAACA
gst-8	CGATCGCTGATCAGTTCAAA	CTGAGCAATGAGCAAATCCA
gst-9	CAAGTTGATGCTCTCGGTGA	GGCCAAATCCATCCAAGTTA
gst-12	CCGGAAAATCAGTGGAAGAA	AGCTGCAATTTTTGGTTGCT
gst-20	TTTTAACGGACGAGGATTGG	GATTTGGAGTCCCGAACTGA
gst-21	TGACATGAAAACGGGATTGA	CCGCTTGAGCTTCCTCTATG
gst-24	GAAATCGAGCGAATCCAAAAG	CTGCAAGCTTCTGCTCCTCTC
gst-25	ATGGACTCAACGGAACCAAC	TCAAGACGTGCGAGATGTTC
gst-30	CCCATGCAATTTCTCGTTTTC	CTCCTTGTGCAAATCCCATTT
gst-31	CTCTCGATTCTACGCCGAAC	GTTGCAAGGTAGCGGATGAT
gst-35	ATTTGGAAGATTCCCGATCC	TCCGGTTTTCCACTTTTCTG
gst-38	CGGAGGCTCTTTACACAAGC	CTTTTCACGTCCCAGCAAAT
skn-1	CTCCATTCGGTAGAGGACCA	GGCGCTACTGTCGATTTCTC
gst-4	TGCTCAATGTGCCTTACGAG	AGTTTTTCCAGCGAGTCCAA
gst-7	AATTCGTGGAGCTGGAGAGAT	CAGCAACCGAGTTGACTTGA
gst-10	ATTCGAAGACATTCGGTTCG	AACATGTCGAGGAAGGTTGC
gstk-1	GGTGTTAAGAATCCGGAGCA	AGAAGGTCGGCAATCAAATG
gpx-7	GTCGACGGGGACAATTTATG	AATTGGTTGCATGGAAAAGC
